# Erectile Dysfunction Following Intravitreal Bevacizumab

**DOI:** 10.4103/0974-9233.65497

**Published:** 2010

**Authors:** Jayshan Yohendran, Devinder Chauhan

**Affiliations:** Vision Retinal Institute, 852 Whitehorse Road, Box Hill VIC 3128, Australia

**Keywords:** Antivascular Endothelial Growth Factor Agents, Bevacizumab, Erectile Dysfunction

## Abstract

Despite initial concerns regarding systemic complications, the use of intravitreal antivascular endothelial growth factor (anti-VEGF) agents for ocular disease is rapidly expanding worldwide, in terms of both the number of patients injected and its indications. To our knowledge, there are no cases in the literature reporting erectile dysfunction following the use of intravitreal bevacizumab. We postulate an organic mechanism for impaired erectile function due to systemically absorbed intravitreal bevacizumab. We describe a case of erectile dysfunction following intravitreal bevacizumab administration. Color fundus photos, fluorescein angiogram and optical coherence tomography images are presented. A 40-year-old male underwent intravitreal bevacizumab therapy for macular edema secondary to a branch retinal vein occlusion. He subsequently developed transient erectile dysfunction after each of his two bevacizumab injections. His only comorbidity was mild hypertension. Erectile dysfunction may be a side effect of intravitreal bevacizumab. The erectile dysfunction could be organic and/or psychogenic in etiology.

## INTRODUCTION

Despite initial concerns regarding systemic complications, the use of intravitreal antivascular endothelial growth factor (anti-VEGF) agents for ocular disease is rapidly expanding worldwide, in terms of both the number of patients injected and its indications. We report the first case of erectile dysfunction following the use of intravitreal bevacizumab. A mechanism is postulated in which systemically absorbed bevacizumab inhibits VEGF in the corpus cavernosum and interacts with nitric oxide to impair erectile function.

## CASE REPORT

A 40-year-old male presented with a three-week history of painless loss of vision in his right eye, without any systemic symptoms. He had no history of cardiac disease or diabetes mellitus, and was a nonsmoker. His general practitioner had been monitoring his mild hypertension for several months, but had not begun treatment; his blood pressure was 135/90mmHg on presentation. He had no other risk factors and no past ocular history of note.

**Figure 1:**
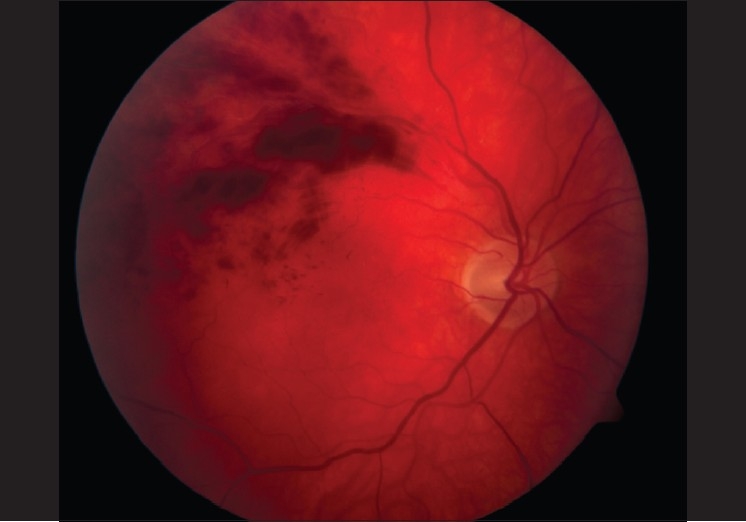
Right eye color fundus photo showing multiple intraretinal hemorrhages peripheral to an arteriovenous crossing site causing venous obstruction

**Figure 2:**
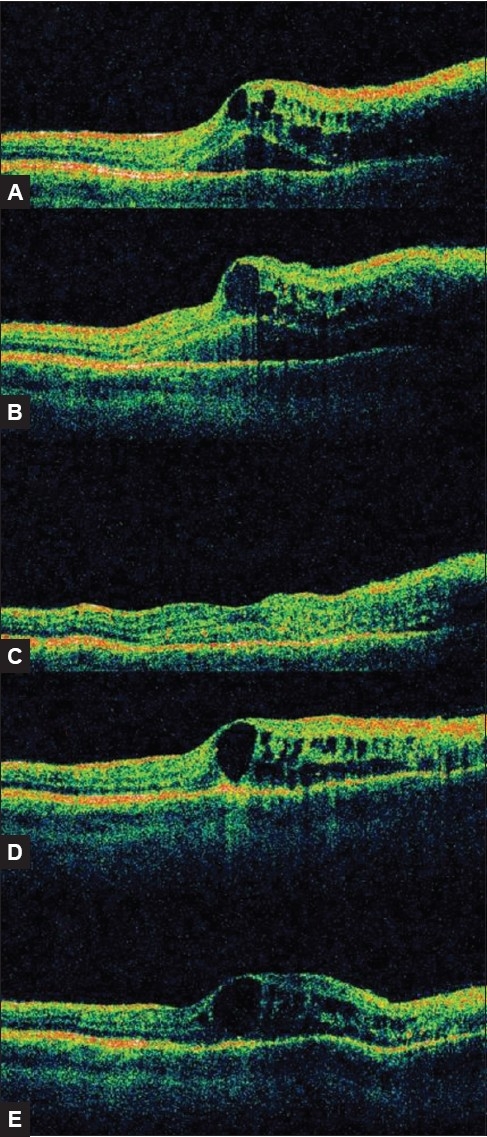
OCT scan images of the right macula, A) at presentation, B) 3/12 after presentation, just prior to first intravitreal bevacizumab injection, C) 2/52 post first intravitreal bevacizumab injection, D) 6/12 after presentation, just prior to second intravitreal bevacizumab injection, E) 2/52 post second intravitreal bevacizumab injection. All OCT scans were taken in the same scale and orientation (vertically and passing through the fovea)

His visual acuity was 6/15 (right eye) and 6/5 (left eye). Fundus examination of the right eye revealed a right superotemporal branch retinal vein occlusion (BRVO). There were multiple intraretinal hemorrhages peripheral to a point of arteriovenous nicking [Figure 1] , with associated retinal edema, demonstrated by optical coherence tomography (OCT) [Figure 2A]. The left eye was unremarkable.

The following serological test results were normal: full blood count, electrolytes, urea, creatinine, liver function, ESR, C-reactive protein, fasting glucose, lipid studies. No thrombophilia was found. His general practitioner started antihypertensive medication at this stage.

**Figure 3:**
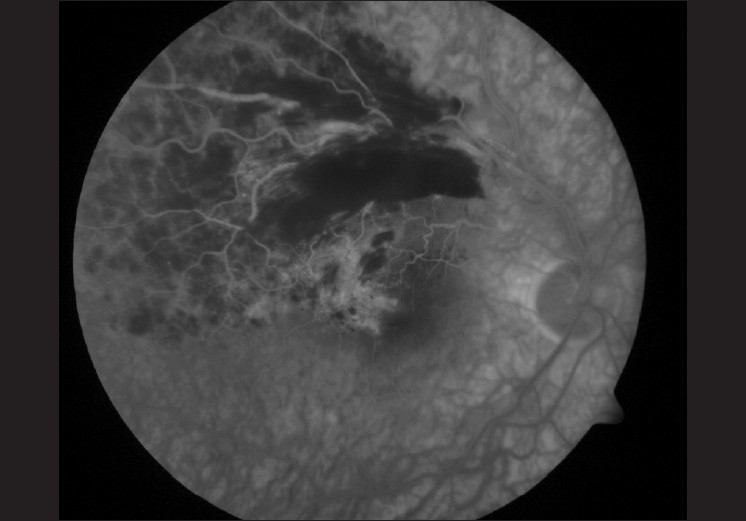
Right eye, late phase of a fluorescein angiogram showing leakage at the macula

He returned six weeks later with further diminished vision in the right eye to 6/18 and an increase in retinal thickness [Figure 2]. A fluorescein angiogram demonstrated the retinal vein occlusion to be ischemic, with some disruption of the perifoveal capillary ring and leakage at the center of the macula [Figure 3]. Treatment options, including observation alone, laser photocoagulation, intravitreal triamcinolone and intravitreal bevacizumab, were discussed with the patient. Laser treatment was precluded by the presence of extensive retinal hemorrhage and gross macular edema. Intravitreal bevacizumab was administered approximately three months after the original diagnosis was made. The patient was fully informed about the novel and off-label character of the treatment as well as the potential risk to his cardiovascular system.[1] The administration of the intravitreal bevacizumab (1.25mg/0.05mL) via the pars plana using an aseptic technique was uncomplicated.

The patient was reviewed three days later, earlier than planned, having reported erectile dysfunction. This was most marked on the night of the injection of bevacizumab. He had been unable to achieve a penile erection that was satisfactory for sexual performance but was, however, able to reach orgasm and ejaculation. This returned to normal gradually within one week. He had no prior history of erectile dysfunction.

His visual acuity on the right had improved to 6/12 two weeks after the intravitreal bevacizumab injection and OCT demonstrated diminution of the retinal edema [Figure 2C]. It was decided to apply grid pattern laser photocoagulation on this day in an attempt to prolong the improvement with bevacizumab and reduce the need for further intravitreal injections.

Three weeks later, there was further reduction in retinal thickness on OCT, but this was transient. The retinal thickness increased and vision deteriorated eight weeks after the laser photocoagulation to 6/36 and, following lengthy discussion, the patient elected to have a further intravitreal injection of bevacizumab. This had lessened effect on his retina as well as a reduced, but not absent, effect on erectile function; his vision only improved to 6/21.

## DISCUSSION

Macular edema is the most common cause of visual loss, in BRVO. There is an ever-increasing number of cases in the literature describing the efficacy of bevacizumab for the treatment of macular edema due to BRVO.[2] Bevacizumab is an agent that inhibits the effects of all isoforms of vascular endothelial growth factor (VEGF).[3] As yet, to the authors’ knowledge, there are no reported cases of erectile dysfunction as a side effect of intravitreal bevacizumab.

Penile erection is a neurovascular phenomenon that involves three synergistic and simultaneous processes: 1) neurologically mediated increase in arterial flow, 2) relaxation of cavernosal smooth muscle, and 3) restriction of venous outflow from the penis. The corpus cavernosum of the penis is composed of a meshwork of interconnected smooth muscle cells lined by vascular endothelium.[4]

Erectile function is dependent on the release of nitric oxide (NO) from nerve endings and vascular endothelium and consequent smooth muscle relaxation with vascular engorgement. The concentrations of NO are controlled predominantly by endothelial nitric oxide synthase (eNOS). VEGF upregulates eNOS in corpus cavernosal smooth muscle cells and elicits an increase in production of NO in human endothelial cells grown in culture.[5] Intravenous injection of VEGF is reported to facilitate the recovery of erectile function in a rat model of arteriogenic erectile dysfunction.[6] Blocking the effect of VEGF could conceivably have the opposite effect, inhibiting erectile function.

Recent work by Bakri *et al*, using a rabbit model, has show that after intravitreal injection of bevacizumab, low concentrations were detectable in the serum.[7] The peak serum concentration was detected eight days after intravitreal injection in the rat model. However, the rat retina is less vascular than the human, which may alter the pharmacokinetic findings in humans.

Erectile dysfunction can be broadly classified as organic or psychogenic, with the majority of cases having a multifactorial etiology. We believe that the administration of intravitreal bevacizumab caused the transient erectile dysfunction in our forty-year-old patient. The trauma of the intravitreal injection might have caused a psychogenic component of his erectile dysfunction; however, an organic cause is postulated. A plausible organic mechanism is through systemic absorption of bevacizumab and the inhibition of VEGF in the corpus cavernosum. The resulting lack of NO production and release might have impaired cavernosal smooth muscle relaxation and arterial flow. Thus erection was not achieved. The lesser effect on erectile function, as well as on the retina, of the second injection may be related to reflux of the injected drug at the time of injection, or possibly tachyphylaxis.

The same side effect might be expected with ranibizumab, but this may be limited due to its lower serum concentration following intravitreal injection and its shorter systemic half-life when compared with bevacizumab.[8] There is no mention of erectile dysfunction in any of the prospective, randomized clinical trials with this drug.[9,10] This may be due to either the absence of the side effect or the lack of recognition and/or reporting of erectile dysfunction in the elderly cohorts studied.

This case highlights the potential systemic side effects of intravitreal anti-VEGF medications, particularly at a time when the number of intravitreal anti-VEGF treatments is rising rapidly, the indications are expanding and patients are undergoing several repeat injections.
